# The Stakeholders’ Views on Planting Trees to Control Schistosomiasis in China

**DOI:** 10.3390/ijerph17030939

**Published:** 2020-02-03

**Authors:** Jun Yang, Jinxing Zhou, Jing Jin, Qixiang Sun

**Affiliations:** 1 Department of Earth System Science, Tsinghua University, Beijing 100084, China; jinj15@mails.tsinghua.edu.cn; 2 Ministry of Education Field Research Station for East Asian Migratory Birds and Their Habitats, Tsinghua University, Beijing 100084, China; 3 School of Soil and Water Conservation, Institute of Forest Ecological Engineering for Schistosomiasis Control, Beijing Forestry University, Key Laboratory of State Forestry Administration on Soil and Water Conservation, Beijing 100083, China; Zjx9277@126.com; 4 Research Institute of Forestry, Chinese Academy of Forestry, Beijing 100091, China

**Keywords:** schistosomiasis, control, tree planting, impact, environment, poverty

## Abstract

China has initiated a tree planting program in epidemic regions of schistosomiasis as a part of efforts to eliminate schistosomiasis. More than 518,900 ha of tree plantations have been planted through the program between 2006 and 2015. However, whether the planting program has fulfilled its mission or not is an open question. In this study, we intended to get the answer from the main stakeholders of the planting program through a large-scale survey. Based on interviews with 80 administrators of the planting program and 1440 farmers in 24 counties and districts in four provinces, we found that most stakeholders viewed the planting program positively. Nearly 92% of farmers and all administrators believed that the planting program had reduced snail densities, while 94.3% of farmers and all administrators believed that the program had lowered the incidences of schistosomiasis. In addition, they reported that the impacts on farmers’ living and local environments by the tree planting program were mainly positive. Based on the stakeholders’ responses, we conclude that the tree planting program has been perceived by the main stakeholders as an effective environmental control measure of schistosomiasis. However, certain places and people that may be impacted negatively by the program should be given more attention when implementing the program.

## 1. Introduction

Schistosomiasis is a parasitic disease that affects millions of people worldwide. According to the World Health Organization (WHO), schistosomiasis was reported in 78 countries, and 220.8 million people required preventive treatment in 2017 [1]. People who are socioeconomically disadvantaged in the epidemic regions are often impacted by the disease the most. Patients are further dragged into poverty as they are burdened with the treatment cost and the loss of labor ability. Due to its destructive impacts, the elimination of schistosomiasis was set by the international community as a target in the resolution of the 65th session of the World Health Assembly [2]. The sustainable development goals also have a specific target on the neglected tropical diseases, which include schistosomiasis [3].

Currently, the WHO is leading a preventive chemotherapy campaign globally to control schistosomiasis. However, to eliminate schistosomiasis requires effective means to stop the transmission of the parasites (Schistosoma) besides chemotherapy [4]. People and animals get infected when they are exposed to water contaminated by the cercariae of the parasites (e.g., *Schistosoma japonicum*). Before that, the parasites infect certain types of freshwater snails (e.g., *Oncomelania hupensis*) as miracidia hatched from eggs contained in feces of patients and sick animals [5]. Various control measures have been used to cut off this transmission pathway. Spaying molluscicides in snail habitats to kill snails has been practiced widely [6,7]. In addition, chemicals have been used to kill cercariae in the water [8,9]. Other than the chemical control measures, environmental control measures that aim to make snail habitats less habitable have also been widely used [10]. For example, soil moisture can affect the snail‘s survival and growth [11,12]. Therefore, measures such as increasing the soil drainage and sealing the soil surfaces of the ditches and streams with concrete have been utilized to lower the snail density in the infected area [13,14]. A more restrictive control measure is to keep people and animals out of waters and waterside areas infected with parasites. Those controlling measures are seldom used separately; instead, they are applied as an integrated control package [15,16]. They have helped to bring the schistosomiasis epidemic under control in some countries. For example, the number of schistosomiasis patients and acute cases have been reduced by 85.5% and 99.7% in China between 2005 and 2014 [17].

While significant progress has been made in controlling the transmission of schistosomiasis, some issues also surfaced during this process. Applying chemicals to kill snails and cercariae carries the risk of increasing chemical resistance of the vector and the parasite [18] and the collateral kill of beneficial organisms [19]. Environmental control measures avoid this problem, but the financial resource needed for initiating and sustaining the environmental control measures can become a burden to local governments. For example, in Fuqing City, Fujian Province, China, the government’s spending on environmental control measures accounted for 65% of the total budget of schistosomiasis control between 2006 and 2010 [20]. To deny people and livestock access to infected areas can create challenges in management. On the one hand, farmers who use infected areas for farming or raising animals tend to ignore the ban due to traditions or economic reasons. On the other hand, local governments have to find resources to enforce the ban and to compensate affected farmers [21].

An environmental control measure that was officially started in China in 2006 has the potential to address these limitations. The government supports local farmers and private enterprises to grow tree plantations in epidemic zones of schistosomiasis in China. Species used for planting usually are fast-growing timber or pulp tree species such as poplars (*Populous* spp.) or Chinese wingnut (*Pterocarya stenoptera*). The site-preparing activities and growth of trees can change the microclimates and soil moisture conditions in snail habitats. Researchers observed the lowered water table, changed light conditions, and restricted growth of weeds in plantations in field experiments [22]. These changes have contributed to the reduction of the snail density. Zero snail density has been observed in plantations that were 10–15 years old, while the significantly lowered snail density was observed in young plantations compared with that of the controlled sites [22,23]. In addition, the planting program helped to keep people and domestic animals away from infected areas [24]. Owners of plantations are working diligently to bar people and animals from their plantations to avoid damages on trees. They inadvertently relieve the burden on the government to enforce the ban. Most importantly, the planting program provides an alternative income source to local farmers. By selling timbers and non-timber products or being employed by owners of tree plantations, farmers can make up the economic loss due to the cessation of farming and grazing activities in the infected areas. The financial gain helps to reduce famers’ needs to use infected areas to make a living.

The central part of the tree planting program has been completed between 2006 and 2015. Around 518,900 ha of tree plantations have been planted in seven provinces through this program [25]. The program was believed to lower snail densities and incidences of acute schistosomiasis diseases in the epidemic zones [24,25,26]. However, the declining trends of the disease and vector populations reported by the existing studies were based on the monitoring data released by the Center for Disease Prevention and Control of China (China CDC). The data do not separate the influences of the planting program from other controlling measures. The economic and ecological benefits of the planting program reported in the existing literature were mainly obtained by extrapolating values measured in other places to studied areas [26,27]. These estimates contain high uncertainties. So far, the effectiveness of the planting program has not been independently confirmed. Furthermore, a question that has been left out by existing studies is: are there negative impacts associated with the planting program? There are concerns that growing plantations along the river banks and lakesides may disturb wildlife habitats and increase flood hazards [28]. To determine whether the tree planting program is a genuinely useful environmental measure for controlling schistosomiasis, we need studies that address the aforementioned questions and concerns.

In this study, we intend to answer the aforementioned questions through face-to-face interviews with the main stakeholders of the program: administrators of the planting program and local farmers. Specifically, we wanted to find out: (1) What were the stakeholders’ views on the effectiveness of the program to lower the snail density and the incidence of the schistosomiasis? (2) How did they view the impacts of the program on farmers’ living and the environment? (3) What factors would affect farmers’ participation in the planting program? Through the study, we found that the planting program was generally viewed as an effective way to control snails and reduce diseases. It had positive impacts on farmers’ incomes and local environments. The financial statuses of farmers affected their participation in the program significantly. In the rest of the paper, we presented the details of our study and the main findings.

## 2. Materials and Methods

### 2.1. Study Area

**Figure 1:**
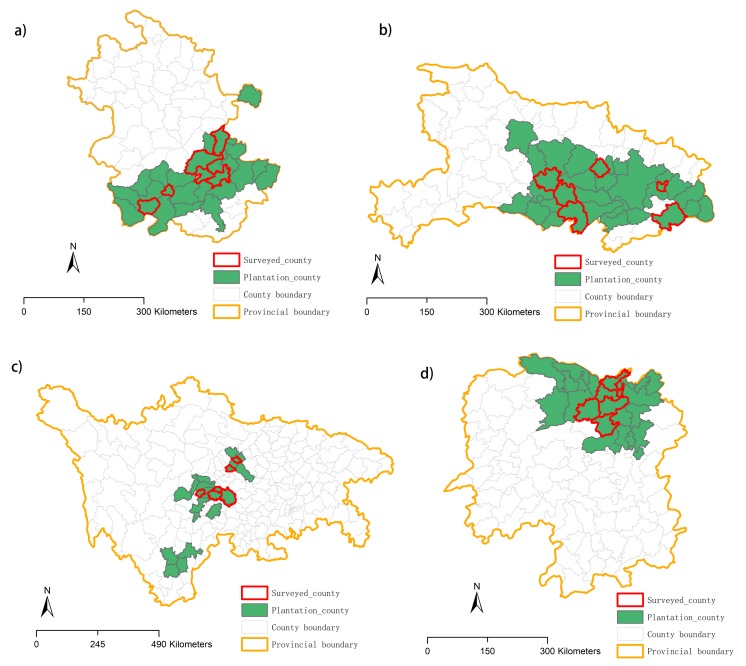
Study area. (**a**) Anhui province, (**b**) Hunan province, (**c**) Sichuan Province, and (**d**) Hubei Province.

We chose Hunan Province, Hubei Province, Anhui Province, and Sichuan Province as the study area. All four provinces have been classified as schistosomiasis epidemic zones by the Chinese health administration. By the end of 2015, 408,559 ha of tree plantations have been planted by the program in these provinces, accounting for 73.8% of the total areas planted nationally. We selected six counties or districts that have implemented the planting program from each province to survey (Figure 1).

### 2.2. Design of Survey Questions

Two questionnaires consisting of multiple-choice and open-end questions were designed in this study: one for administrators and one for local farmers. The interview questions for the administrators included three sections. In the first section, administrators were asked about their positions and knowledge of schistosomiasis. In the second section, they were asked questions on the primary control measures of schistosomiasis used in their counties or districts. Questions on the effectiveness of the tree planting program were asked in the third section. Administrators were asked to assess the impacts of the planting program on snails, schistosomiasis, the environment, and local farmers’ incomes.

Interview questions for farmers also included three sections. Demographic and socioeconomic information about the participants was collected in the first section. In the second section, the participants were asked about their knowledge of schistosomiasis and controlling measures. In the third section, the participants were asked questions about the planting practices and the effectiveness of the planting program.

We tested the two questionnaires in four counties in Sichuan Province before the formal survey. These counties were different from the counties included in the formal survey to avoid biases. Based on the responses from the investigators and participants in the pilot study, we modified the questionnaires to remove questions that could lead to ambiguous or uncertain answers. The final questionnaire for administrators had 11 questions, while the final questionnaire for local farmers contained 18 questions (see Table S1 and Table S2 in the Supplementary Files for the questionnaires). We prepared detailed instructions on how to fill the questionnaire and trained the investigators—graduate students in the School of Soil and Water Conservation, Beijing Forestry University—before running the formal survey.

### 2.3. Data Collection and Analysis

In order to gain a general understanding of the tree planting program in the study area, we obtained the statistics on acreages of trees planted by the program in the studied area between 2006 and 2015 from the State Forestry Administration of China. Data on areas that were classified as habitats for snails were obtained from the Chinese CDC.

In each county or district, we randomly selected 60 families in villages where the planting program has been implemented to conduct interviews. In addition, we interviewed three administrators of the planting program in each county or district and two administrators at the provincial level in each province. They were selected from a list of administrators who were directly involved in the planting program. All interviews were conducted in a face-to-face manner, with one investigator serving as the principal questioner and another one serving as recorder.

After surveys, we conducted a quality check and discarded forms with uncompleted information. Using the validated forms, we summarized the responses on three major topics: the respondents’ knowledge of schistosomiasis control, the field practices of tree planting by local farmers, and the views on the impacts of the tree planting program. Then, we compared responses to six questions between the administrators and the farmers (Table 1).

To find out how the family condition and knowledge of schistosomiasis control affect a farmer’s participation in the planting program, we fitted a generalized linear mixed model (GLMM) using the survey data of farmers. The family’s participation in the planting program was treated as the response variable, which has values of 1 (participated) and 0 (did not participate). The number of labors, family incomes, knowledge of schistosomiasis, participation in other controlling measures, and the use of lands prior to tree planting was treated as explanatory variables (Table 2). Since the response variable was a binary variable, we fitted a binomial model and used the logit function as the link function. The counties or districts were fitted as the random effect.

## 3. Results

### 3.1. The Planted Areas and the Overall Trend of Snail Habitats

Between 2006 and 2015, a total of 143,294.7 ha of tree plantations have been planted in these counties and districts. The average area of plantation planted in a county or district was 5970.6 ha (276.7–41,200 ha). Snail habitats in 17 counties and districts have declined, while an opposite trend was observed in seven counties and districts (Table 3). The Pearson correlation coefficient between the area of plantation and changes of snail habitats was -0.17 (*t* = -0.809, *df* = 22, *p*-value = 0.4271).

### 3.2. Characteristics of Respondents

In total, we interviewed 80 administrators and 1440 local farmers in 24 counties and districts. After the quality check, we kept all 80 forms for administrators and 1218 forms for local farmers for further analysis. Among the 1218 local farmers, 707 has participated in the planting program. The demographic and socioeconomic characteristics of the local farmers were summarized as follows (Table 4). The number of male participants was much higher than that of female participants because men often act as the heads of families and are responsible for receiving visitors in the rural area.

### 3.3. Knowledge of Schistosomiasis

Among administrators, 42.5% indicated that they were very familiar with schistosomiasis, while 33.75%, 21.25%, and 2.5% indicated they were familiar, relatively familiar, and less familiar with schistosomiasis, respectively. Among local farmers, 59.52% of respondents could correctly identify all five prevention measures of schistosomiasis. The percentages of farmers that could correctly identify four, three, two, and one prevention measures were 20.03%, 13.05%, 6.07%, and 1.31%, respectively.

### 3.4. Effectiveness of the Planting Program

**Figure 2:**
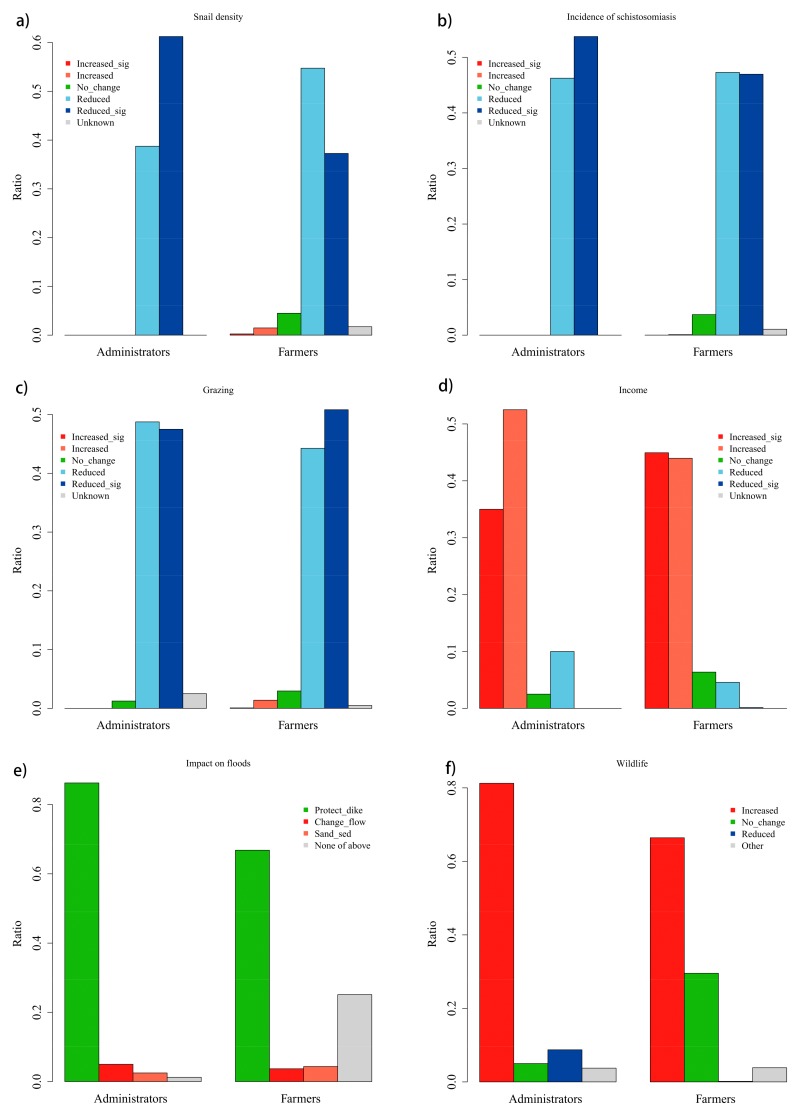
Views on the effectiveness of the planting program. (**a**) Impacts on snail densities, (**b**) Impacts on incidences of schistosomiasis, (**c**) Impacts on grazing, (**d**) Impacts on farmers’ income, (**e**) Impacts on floods, and (**f**) Impacts on wildlife. The abbreviations are sig=significantly, sed=sedimentation.

The administrators and local farmers primarily expressed similar attitudes toward the effectiveness of the tree planting program (Figure 2).

The farmers’ answers to the question regarding the primary benefit and issues of the planting program concentrated on several items (Table 5). Fewer people (124) gave opinions on disadvantages than people who commented on the benefits of trees (1005). Other than the top 10 benefits and disadvantages citied in Table 5, other comments such as “increase the feeling of safety”, “provide fuel”, and “block flood path” received fewer cites. The complete list can be found in the Supplementary File (Table S3).

### 3.5. Factors Affecting Participation in the Planting Program

The result of the GLMM model showed that the family income and the farmer’s belief that plantation could effectively reduce grazing significantly influenced a farmer’s participation in the planting program (Table 6). The variance caused by the random effect was 1.739 ± 1.139 (std). The conditional R^2^ (random effect) was 0.662, while the marginal R^2^ (fixed effect) was 0.08. All these indicated that the odds of participating in the planting program was affected more by the county than by other explanatory variables.

**Figure 3:**
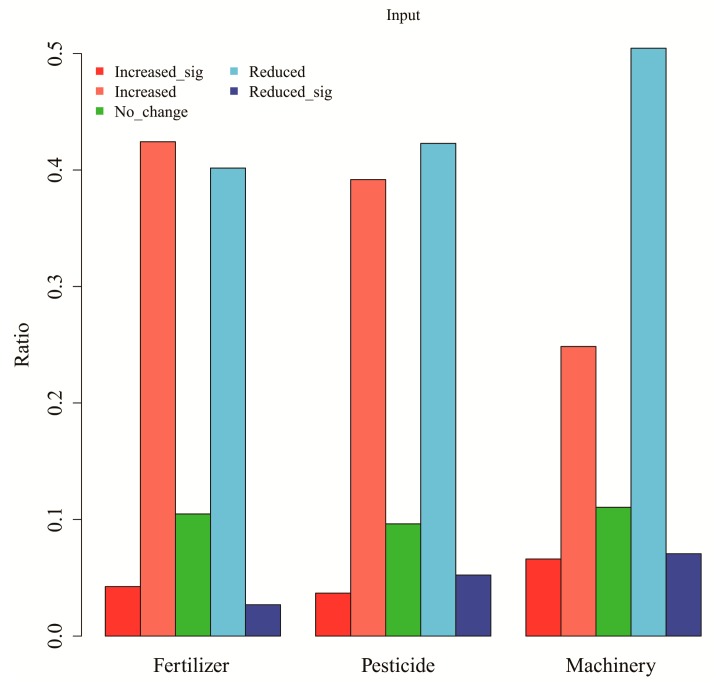
Changes of inputs by famers who have participated in the planting program. The abbreviations are sig = significantly.

The changed inputs in fertilizers, pesticides, and use of machinery reported by farmers who have participated in the planting program showed a mixed pattern (Figure 3).

## 4. Discussion

Snail habitats in most counties and districts have declined between 2006 and 2015. There were seven counties and districts that had the opposite trend. A close look showed that the adjustment of administrative boundaries of these counties and districts during this period was the main reason for this countertrend. Villages or townships with areas infected with snails were later put under their administration. The correlation coefficient indicated a negative but not statistically significant association. The lack of significant association between the areas of plantations and changes in snail habitats was expected because the program was only part of an integrative package of measures used to control snails. The finding supported our early suggestion that it is inappropriate to simply attribute the declination of snails or incidences of disease in these counties or districts to the planting program.

Our results showed that the participants in our survey had a good knowledge of schistosomiasis diseases. Around 76% of administrators were very familiar and familiar with the matter, while around 80% of local farmers had good knowledge of disease prevention. This result showed that the education and outreach program on schistosomiasis has achieved good results in these counties and districts. This observation supported an early finding that health education on schistosomiasis prevention in China is effective [29]. It also added confidence to our results as the interviewees had the knowledge on the topics covered in our survey.

The majority of administrators and farmers believed that the planting program has helped to lower snail densities and incidences of schistosomiasis diseases. The farmers’ answers regarding the main benefit of the planting program further corroborated this point. “Reduce disease” and “Reduce snails” were ranked as the third and the fourth among all citied primary benefits. Lowering snail densities and incidences of schistosomiasis are two main justifications proclaimed for the planting program [24]. The perceived reduction of snail densities by tree planting is supported by results from field studies [22,23]. The percentage of administrators who believed that the program had reduced the vectors and the incidence of disease significantly was higher than that of farmers. This difference could be due to the administrators’ role in promoting the planting program, which made them viewing the program more favorably. It could also be due to the fact that the administrators’ observations reflected the general situation in the county or the province, while farmers’ observations tended to focus on changes in a particular village.

In addition, the majority of the administrators and farmers believed that the planting program has reduced grazing in the infected areas. This observation was further verified by the farmers, as they ranked “Difficult for grazing” the third place among main problems caused by the planting program to farmers. Raising livestock in the infected areas, especially buffalos, constitutes a significant contributor to the epidemic of schistosomiasis in China [16]. The tree planting program has helped to cut this transmission pathway. Stopping grazing and other farming activities in the infected area might affect some farmers’ income. However, the majority of the administrators and farmers found that the planting program had increased farmers’ incomes. The farmers listed “increase income” as one of the main benefits of the program, second only to “improve the environment”. This observation proved that the planting program could provide a way to compensate the farmers financially when they gave up grazing and farming in the infected areas. Past schistosomiasis control programs have been criticized for not considering the economic factors underlying multi-parasite transmission [30]. The tree planting program obviously took the note.

On the ecological impacts of the planting program, the administrators again held a more favorable view than the farmers. More than 80% of administrators believed that the main impact on floods was to protect dikes, while only more than 60% of farmers had the same view. The view on the impact on wildlife showed a similar pattern. Furthermore, more than 20% of farmers did not select any of the three impacts of the tree planting program on floods. The same number of farmers did not think that the planting program has caused changes in wildlife. These divergences can be caused by the different availability of information to administrators and farmers. It could also be caused by the lack of professional knowledge needed for judging these benefits among farmers, e.g., wildlife identification skills and knowledge of flood engineering. A small percentage of administrators and farmers reported that tree planting could affect the flood flow path and caused sand sedimentation. However, a field study found that poplar plantations actually caused less sedimentation than reeds [31], which was the original vegetation type in most planting sites. Some administrators reported reduced wildlife in planting sites. Studies on the impact of plantations on biodiversity did not report a uniform trend, with the increase of plant diversity [32] and the decrease of bird [33] and insect diversity [34] being reported at the same time. More studies will be needed to look into these impacts.

The main benefits and problems of the program expressed by the farmers were mostly in agreement with their choices above. One notifiable point is that farmers listed “Improve the environment” as the top benefit and ranked “Improve air quality” in the fifth place. These are co-benefits of the planting program. Several studies have shown that tree plantations planted through the program can help to improve environments and clean air [26,27]. These benefits were citied more by the farmers than “Reduce snails”, which was probably because the change in greenery was more observable than changes in snail densities.

The GLMM fitting result showed that the random-effects variance (between counties) was more than the fixed-effects variance. This was understandable because each county made their own policies to decide who could participate in the planting program and the level of subsidization given to participants. However, two factors were significant at the individual level, i.e., the annual family income and the perception of tree plantations as the most effective way to control grazing in the infected area. Growing tree plantations requires investments in tree seedlings, fertilizers, pesticides, and management. Comparing to conventional farming, the upfront cost of tree planting was much higher. In addition, due to the long growing time of trees, farmers cannot get immediate returns. These factors were displayed in farmers’ ranking of the main problems of the program. “Reduce income or slow return” and “Need investment” were ranked as the top and the fifth place, respectively. There is a danger that the planting program may favor families in better economic situations more than poor families. Especially when the poor families relied on the land for incomes, the program might deepen the income inequality by denying them the access to the land. Other large-scale afforestation projects have already been found to contribute to income inequality in rural China [35]. This is an issue that need to be concerned by the government. The significant influence of the perception of tree plantations as the most effective way to control grazing in the infected area on farmers’ decision to plant trees might be a self-enforced belief. Farmers who have participated in the planting program might have a strong belief in its effectiveness.

The number of labors in a family did not have a significant effect on farmers’ participation in the planting program. This indicated that the program would not increase labor demands from the participated families significantly. In contrary, “Lower labor intensity” was ranked the sixth among all benefits. Knowledge of schistosomiasis and participation in other controlling measures also did not affect the participation significantly. This might suggest that farmers’ participation in the planting program was more from a utilitarian view rather than a disease prevention view. The negative association between the participation in the program and the number of use types of the lands for other purposes, even though not statistically significant, added supports to this observation. All these observations attested to the importance of economic factors in schistosomiasis control [30].

The changes of agricultural inputs by farmers who have participated in the program showed a mixed pattern. Almost an equal number of farmers reported increased or decreased use of fertilizers and pesticides. This seemly contradicted pattern reflected the diverse uses of the lands before planting trees. For families that used the lands for low-intensity production activities such as for animal grazing, growing tree plantations would increase their inputs. However, for those who used the lands for more extensive farming activities such as growing crops and vegetables, conversion to tree plantations could lower the inputs of fertilizers and pesticides. The more uniform opinion that the tree planting program could reduce the use of machinery was due to the fact that machinery was less needed when trees grow up.

In summary, by surveying the main stakeholders of the tree planting program, we found that their responses support the justifications used to initiate the program. The program has been perceived to reduce snail densities, incidences of schistosomiasis, and grazing in the infected areas. While achieving these goals, the program was reported to have mostly positive impacts on the farmers’ income and environments. However, our study also revealed some impacts that require more attention. The negative impacts on floods and wildlife observed by some stakeholders need to be further studied. The significant influence of the annual family income on participation in the program brought the concern on whether the program will increase income inequality in rural areas. While these are not significant issues for the program as a whole, they might cause adverse impacts to certain groups of people and planting sites.

As the first large-scale study on the effectiveness of the tree planting program, our study has provided vital information to gauge the success of the program and its potential inclusion as a tool for controlling schistosomiasis. However, our study has limitations. First, we were only able to conduct a cross-section study. A longitude study tracking the before-and-after changes will be more ideal. We wish that the government will consider setting up a longitudinal study in the next stage of the planting program. Second, we have to rely on the administrators’ and farmers’ observations to assess the impacts of the planting program. This will inadvertently add subjectivity to our results. It will be more rigorous to quantify the impacts of the planting program through controlled studies. Nevertheless, to conduct controlled studies will be a real challenge, because it is unethical to prevent people from using other means to control the vectors and diseases other than planting trees.

## 5. Conclusions

To eliminate schistosomiasis is a health goal set up by the international community. Environmental controlling measures play an essential role in realizing this goal. In this study, we assessed the effectiveness of using the tree planting program to control schistosomiasis in China through a large-scale survey. Our results showed that the majority of stakeholders viewed the tree planting program as a success. The program was credited for lowering snail densities and incidences of schistosomiasis in places where it has been implemented. The program was also found to increase farmers’ incomes and improve environments. While the mainstream view toward the planting program was positive, there were also concerns about its adverse impacts on environments and income inequality in certain places. More attention needs to be paid to these impacts in the future. Overall, our study showed that the tree planting program had addressed a number of limitations associated with the existing environmental control measures. Other countries and regions that face the challenging task of eliminating schistosomiasis may consider adding this control measure to their arsenals.

## Figures and Tables

**Table 1:** Six questions used to compare the differences in attitudes toward the planting program between administrators and farmers.

Variables	Description
Impact of plantations on snail densities	A = “Increased significantly”, B = “Increased”, C = “No change”, D = “Reduced”, E = “Reduced significantly”, F = “Unknown”
Impacts on incidences of schistosomiasis	A = “Increased significantly”, B = “Increased”, C = “No change”, D = “Reduced”, E = “Reduced significantly”, F = “Unknown”
Impacts on grazing	A = “Increased significantly”, B = “Increased”, C = “No change”, D = “Reduced”, E = “Reduced significantly”, F = “Unknown”
Impacts on floods	A = “Protect banks and dikes”, B = “Change the flow paths of floods”, C = “Create sand sedimentation”, D = “None of them”
Impacts on wildlife	A = “Increased”, B = “No change”, C = “Reduced”, D = “Other”
Impact on farmer’s income	A = “Increased significantly”, B = “Increased”, C = “No change”, D = “Reduced”, E = “Reduced significantly”, F = “Unknown”

**Table 2:** Description of explanatory variables used in the generalized linear mixed model (GLMM) model.

Variables	Description
Socioeconomic characteristics	
Number of labors	Number of family members ≥ 18 years old
Family income	Annual household income, with 1 = ”<RMB10K”, 2 = ”RMB10K-29K”, 3 = ”RMB30K-49k”, 4 = ”RMB50K-69k”, 5 = ”≥RMB-70K”
Knowledge and participation	
Knowledge of schistosomiasis	Respondents’ knowledge of schistosomiasis, with 5 = correctly selected all five effective measures; 0 = no effective measures have been correctly selected
Participation in control activities	The number of control measures that respondents have participated in other than tree planting
Most effective measures to control grazing	Respondents’ choice of the most effective measure to control grazing, with 1 = if plantation selected, 0 = plantation not selected
Use of lands	
Types of use	The number of activities practiced at lands other than tree planting

**Table 3:** Total planted areas and changes in acreages of snails’ habitats in 24 counties/districts between 2006 and 2015. The data for areas of tree plantations came from the State Forestry Administration of China while the data for changes in snail habitats came from the Center for Disease Prevention and Control of China (China CDC).

Province	Name of the County/District	Area of Tree Plantation (ha)	Change in the Area of Snail Habitat (%)
Anhui	Daguan District	666.7	+86.4
	Tongling County	1160.0	−12.9
	Nanling County	3626.7	−18.7
	Hexian County	3953.3	+0.04
	Wangjiang County	4880.0	−19.4
	Wuwei County	6376.0	−71.1
Hubei	Yingcheng County	2513.3	−14.5
	Qianjiang County	2715.0	−23.6
	Shayang County	3390.0	−97.0
	Yangxin County	3736.7	−28.7
	Huangzhou District	4053.3	−30.0
	Jianli County	11,393.3	−7.8
Hunan	Datonghu District	1222.1	−4.9
	Heshan District	1366.7	−21.3
	Nanxian County	6666.7	+3.4
	Huarong County	10,000.0	−20.0
	Hanshou County	23,000.0	−0.4
	Yuanjiang County	41,200.0	−0.3
Sichuan	Guanghan City	276.7	+231.8
	Mingshan District	680.3	−100.0
	Luojiang County	1060.0	+401.1
	Pengshan District	1666.0	+1438.1
	Dongpo District	1963.3	+811.2
	Renshou County	5728.7	−71.1

**Table 4:** Summary statistics of the local farmers.

Characteristics	Number	Percentage (%)
Gender		
Female	112	9.2
Male	1106	90.8
Age Class		
<20	2	0.16
20–39	143	11.74
40–59	818	67.16
≥60	255	20.94
Education Level		
Illiterate	24	1.97
Primary School	234	19.21
Middle School	609	50.0
High School	276	22.66
College and Above	75	6.16
Annual Family Income Level		
<10K	38	3.12
10K–29K	191	15.68
30K–49K	331	27.18
50K–69K	302	24.8
≥70K	356	29.22

**Table 5:** Top ten benefits and problems caused by the planting programs according to the farmers.

Main Benefit	Frequency	Main Problem	Frequency
Improve the environment	379	Reduce income or slow return	25
Increase income	320	Affect the yield of crops	20
Reduce disease	319	Difficult for grazing	20
Reduce snails	131	Loss of farmland	11
Improve air quality	109	Need investment	7
Lower labor intensity	29	Reduce fishing activities	7
Soil and water conservation	28	Denial of access to places	6
Good for general health	17	Increase forest fire risk	5
Prevent flood	15	Affect farming activities	4
More tree shade	5	Increase tree pests	3

**Table 6:** Estimated regression parameters, standard errors, *z*-values, and *p*-values for the GLMM model.

	Estimate	SE	*z* Value	Pr(>|*z*|)
Intercept	–0.618	0.629	–0.982	0.326
Number of labors	–0.067	0.067	–0.996	0.319
Family income22	0.806	0.436	1.893	0.058
Family income 23	1.206	0.419	2.876	0.004
Family income 24	0.871	0.425	2.050	0.040
Family income 25	1.583	0.432	3.662	0.000
Knowledge on schistosomiasis	0.067	0.081	0.820	0.412
Participate in other controlling programs	0.048	0.072	0.654	0.513
Plantation as the most effective way to control grazing	0.515	0.157	3.273	0.001
Other use of lands	–0.155	0.094	–1.640	0.101

## Supplementary Materials

The following are available online at https://www.mdpi.com/1660-4601/17/3/939/s1, Table S1: The questionnaire for the administrator, Table S2: The questionnaire for the farmer, Table S3: Data used in the study.
