# Supplementary material for: The Stakeholders’ Views on Planting Trees to Control Schistosomiasis in China
Source: Int J Environ Res Public Health. 2020 Feb 3;17(3):939. doi: 10.3390/ijerph17030939 (PMC7036940; doi:10.3390/ijerph17030939)
Supplement: Supplementary file 1 [file ijerph-17-00939-s001.zip › Supplementaryfile/TableS1Thequestionnairefortheadministrator.pdf]

Table S1. The questionnaire for the administrator

Date: Place: Interviewer 1: Interviewer 2:
**Section 1**
1. Name: Institute:
2. What is your familiarity with the knowledge of schistosomiasis?<br>A. Very familiar B. Familiar C. Relatively familiar D. less familiar E. Unfamiliar
**Section 2**
3. What are the main activities practiced in the sites before planting? (multiple choices)<br>A. Growing crops and vegetables B. Aquaculture C. Poultry farming D. Livestock<br>E. Growing reeds F. Others, please fill in______________________________
4. What is the most effective way to control grazing?<br>A. Planting trees B. Education and warning signs C. Seal surface D. Closure<br>E. Financial compensation F. Replace buffalos with machinery
5. What prevention and control measures have been practiced in your region?<br>A. Examine and treat diseases B. Replace buffalos with machinery C. Seal surface D. Plant trees E. Improve water supply and toilets F. Chemical control G. Nothing
**Section 3**
6. How does the planting program affect snail densities?<br>A. Increased significantly B. Increased C. No change D. Reduced<br>E. Reduced significantly F. Unknown
7. How does the planting program affect the incidence of schistosomiasis?<br>A. Increased significantly B. Increased C. No change D. Reduced<br>E. Reduced significantly F. Unknown
8. How does the planting program affect grazing in the infected area?<br>A. Increased significantly B. Increased C. No change D. Reduced<br>E. Reduced significantly F. Unknown
9. How does the planting program affect floods?<br>A. Protect banks and dikes B. Change the flow paths of floods<br>C. Create sand sedimentation D. None of them
10. How does the planting program affect wildlife?<br>A. Increased B. No change C. Reduced D. Other
11. How does the planting program affect the farmers' income?<br>A. Increased significantly B. Increased C. No change D. Reduced<br>E. Reduced significantly F. Unknown
