# Supplementary material for: The Stakeholders’ Views on Planting Trees to Control Schistosomiasis in China
Source: Int J Environ Res Public Health. 2020 Feb 3;17(3):939. doi: 10.3390/ijerph17030939 (PMC7036940; doi:10.3390/ijerph17030939)
Supplement: Supplementary file 1 [file ijerph-17-00939-s001.zip › Supplementaryfile/TableS2Thequestionnaireforthefarmer.pdf]

Table S2. The questionnaire for the farmer

Date: Place: Interviewer 1: Interviewer 2:
**Section 1**
1. Name: Sex: Age: Education:
2. How many people are in your family________? How many of them are older than 18____?
3. What is the total annual income of your family____________?
**Section 2**
4. Which of the following prevention measures for schistosomiasis is correct?<br>A. Do not wash and bathing in infected water B. Wear rainboots when working in the field C. Do not do open defecation D. Do not let animals grazing in infected areas E. Do not make physical contacts with patients F. Do not consume diseased animal meat G. Do not fishing in infected water
5. What did you use the land for before planting? (multiple choices)<br>A. Growing crops and vegetables B. Aquaculture C. Poultry farming D. Livestock<br>E. Growing reeds F. Others, please fill in______________________________
6. What is the most effective way to control grazing?<br>A. Planting trees B. Education and warning signs C. Seal surface D. Closure<br>E. Financial compensation F. Replace buffalos with machinery
7. What prevention and control measures have you participated?<br>A. Examine and treat diseases B. Replace buffalos with machinery C. Seal surface D. Plant trees E. Improve water supply and toilets F. Chemical control G. Nothing
**Section 3**
8. How does the planting program affect snail densities?<br>A. Increased significantly B. Increased C. No change D. Reduced<br>E. Reduced significantly F. Unknown
9. How does the planting program affect the incidence of schistosomiasis?<br>A. Increased significantly B. Increased C. No change D. Reduced<br>E. Reduced significantly F. Unknown
10. How does the planting program affect grazing in the infected area?<br>A. Increased significantly B. Increased C. No change D. Reduced<br>E. Reduced significantly F. Unknown
11. How does the planting program affect floods?<br>A. Protect banks and dikes B. Change the flow paths of floods<br>C. Create sand sedimentation D. None of them
12. How does the planting program affect wildlife?<br>A. Increased B. No change C. Reduced D. Other
13. What is your use of fertilizers before and after planting trees?<br>A. Increased significantly B. Increased C. No change D. Reduced<br>E. Reduced significantly F. Unknown
14. What is your use of pesticides before and after planting trees?<br>A. Increased significantly B. Increased C. No change D. Reduced<br>E. Reduced significantly F. Unknown
15. What is your use of machinery before and after planting trees?<br>A. Increased significantly B. Increased C. No change D. Reduced

E. Reduced significantly F. Unknown
16. How does the planting program affect your family income?<br>A. Increased significantly B. Increased C. No change D. Reduced<br>E. Reduced significantly F. Unknown
*17. What do you think is the main benefit of the planting program?*_____________
*18. What do you think is the main problem of the planting program?*_____________
